# Extra-Anatomic Jump Graft from the Right Colic Vein: A Novel Technique to Manage Portal Vein Thrombosis in Liver Transplantation

**DOI:** 10.1155/2018/4671828

**Published:** 2018-01-14

**Authors:** Paolo Magistri, Giuseppe Tarantino, Tiziana Olivieri, Annarita Pecchi, Roberto Ballarin, Fabrizio Di Benedetto

**Affiliations:** ^1^Hepato-Pancreato-Biliary Surgery and Liver Transplantation Unit, University of Modena and Reggio Emilia, Modena, Italy; ^2^Department of Medical and Surgical Sciences and Translational Medicine, Sapienza University of Rome, Rome, Italy; ^3^Department of Radiology, University of Modena and Reggio Emilia, Modena, Italy

## Abstract

**Background:**

In the context of cirrhosis, portal vein thrombosis (PVT) is present in 2.1% to 26% of patients. PVT is no longer considered an absolute contraindication for liver transplantation, and nowadays, surgical strategies depend on the extent of PVT. Complete PVT is associated with higher morbidity rates and poor prognosis, while comparable long-term outcomes can be achieved as long as physiological portal inflow is restored.

**Materials and Methods:**

We report our experience with a 45-year-old patient undergoing liver transplant with a PVT (stage III-b). To restore portal vein inflow to the liver, an extra-anatomic jump graft from the right colic vein with donor iliac vein interposition was constructed.

**Results:**

The patient recovered well, with a progressive improvement of the general conditions, and was finally discharged on p.o.d. 14. No anastomotic defects were found at the postoperative CT scan 10 months after the surgery.

**Conclusion:**

Our technical innovation represents a valid and safe alternative to the cavoportal hemitransposition, providing a proper flow restoration and reproducing a physiological setting, while avoiding the complications related to the cavoportal shunt. We believe that the reconstitution of liver portal inflow should be obtained with the most physiological approach possible and considering long-term liver function.

## 1. Introduction

In the context of a cirrhotic liver, portal vein thrombosis (PVT) is present in 2.1% to 26% of patients [1]. Thanks to important technical improvements, PVT is no longer considered an absolute contraindication for liver transplantation (LT), and nowadays, surgical strategies depend on the extent of PVT. As we previously reported, PVT can be classified as follows: type 1, partial PVT in the right or left portal vein (PV) branch; type 2, partial PVT in the main PV alone; type 3, partial PVT in the main PV along with a thrombus in the right or left branch or both (type 3a) and/or a partial PVT in the main PV along with a thrombus in the superior mesenteric vein (SMV) or splenic vein (SV) or both (type 3b); and type 4, complete thrombus occluding the main PV alone (type 4a), with or without the right or left branch (type 4b), and SMV or SV or both (type 4c) [2]. Complete PVT is associated with higher morbidity rates and poor prognosis, while comparable long-term outcomes can be achieved as long as physiological portal inflow is restored [3]. Interestingly, the pathogenesis of PVT in patients with cirrhosis has still not been completely clarified: it might be related to (1) the alterations of the liver architecture, (2) increased vascular resistance, and (3) subsequent slowing of the portal blood flow [1]. Rethrombosis after reconstruction is an early complication that occurs more frequently in the first 7 days after surgery [4]. Conversely, PV stenosis occurs in a later stage, usually after 3 or more months [4]. Recently, we successfully treated a young man affected by end-stage liver disease with grade 3b PVT performing an extra-anatomic jump graft from the right colic vein. We herein report our experience together with a literature review of reproducible intraoperative PVT treatments to clarify the most appropriate approach for such a condition.

## 2. Materials and Methods

### 2.1. Literature Search

A systematic literature search was performed independently by two of the manuscript's authors (GT and TO) using PubMed, EMBASE, Scopus, and the Cochrane Library Central. The search was limited to studies in humans and to those reported in the English language, without any filter set for the year of publication or type of publication.

The following MeSH search headings were used: “portal vein thrombosis” AND “jump graft” AND “liver transplantation” OR “liver transplant.” Extensive cross-checking of the reference lists of all retrieved articles that fulfilled the inclusion criteria further broadened the search. For all of the databases, the last search was run on March 31, 2017.

### 2.2. Study Selection

The same two authors independently screened the titles and abstracts of the primary studies that were identified in the electronic search. Duplicate studies were excluded. The following criteria were set for inclusion in this review: (1) studies reporting outcomes of surgical “physiological” approaches for PVT in LT; (2) studies comparing approaches for PVT; and (3) if more than one study was reported by the same institute, only the most recent or the highest quality study was included. Conversely, studies in which it was impossible to retrieve or calculate data of interest were excluded.

The literature search yielded 56 articles; after duplicates were removed, 29 titles and abstracts were reviewed. Most relevant data and papers are reported in Results and later discussed.

### 2.3. Data Extraction

All relevant texts, tables, and figures were reviewed for data extraction.

Discrepancies between the two reviewers were resolved by consensus discussion or with the opinion of the senior author (FDB).

## 3. Results

### 3.1. Case Report

**Figure 1:**
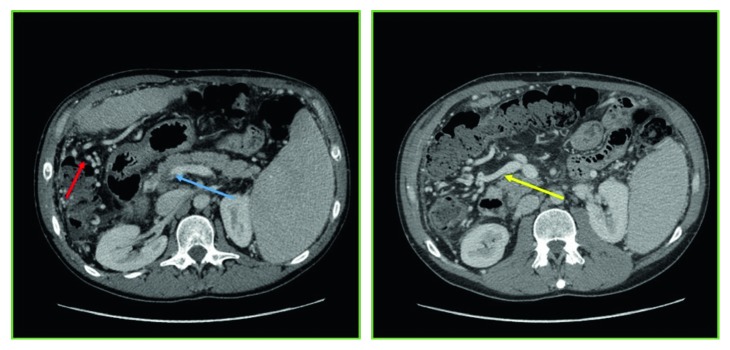
Preoperative CT scan showing PV thrombosis and portosystemic shunts. Blue arrow: PVT. Red arrow: portosystemic shunts. Yellow arrow: right colic vein.

**Figure 2:**
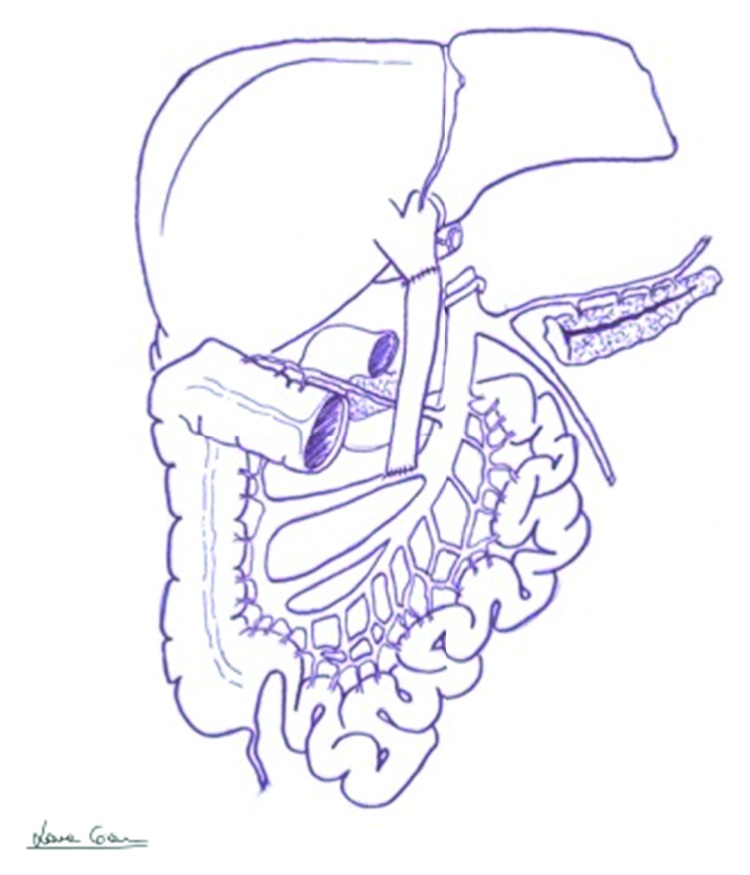
Drawing showing the anatomy after jump graft interposition (the pancreas is split for graphic reasons).

**Figure 3:**
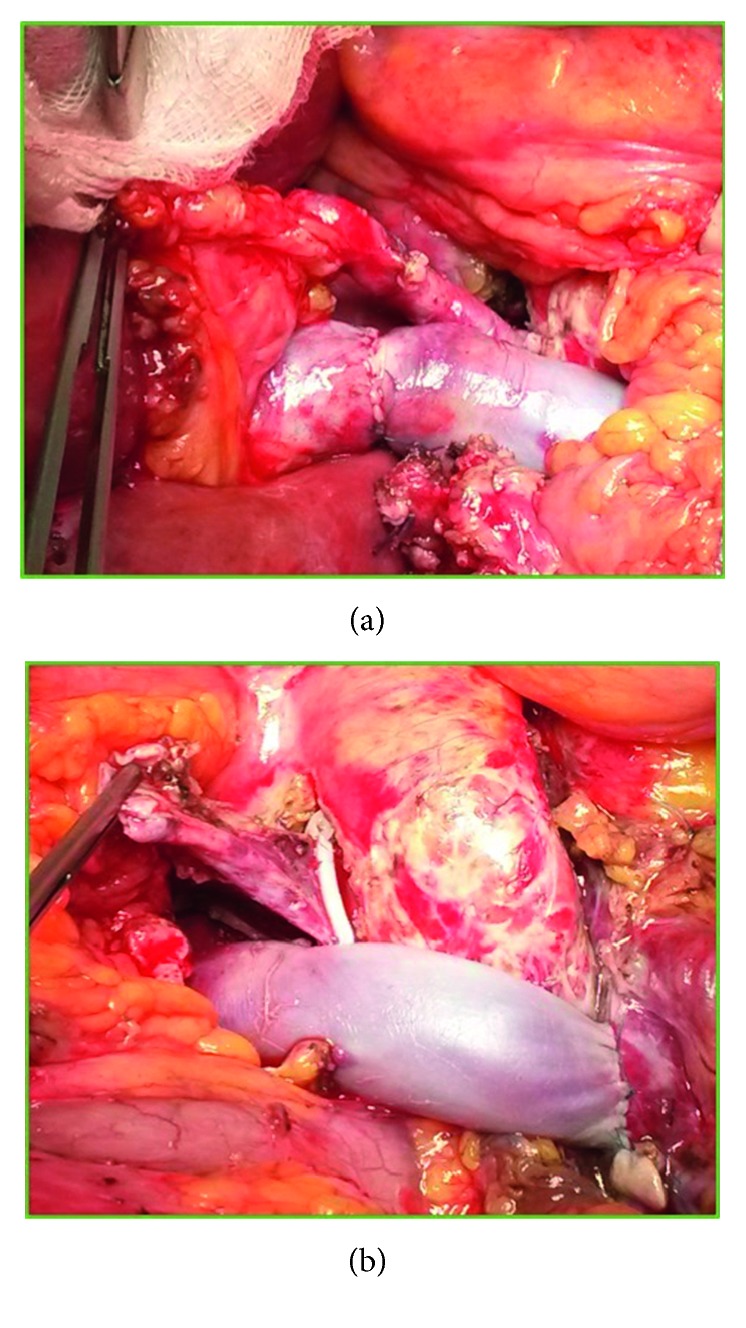
Intraoperative picture showing iliac vein jump graft from the right colic vein to the donor PV. (a) End-to-end PV-jump graft anastomosis; (b) end-to-side jump graft-right colic vein anastomosis. The vessel closed with the Hem-o-lok and lifted with the forceps is the native PV.

**Figure 4:**
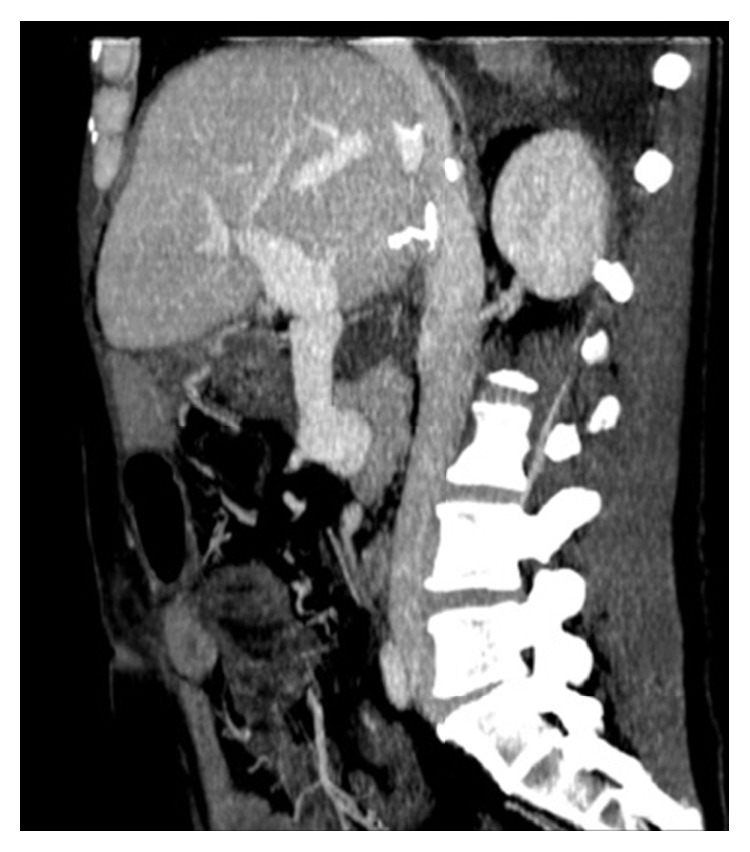
Postoperative CT scan showing regular PV inflow and no anastomotic defects.

We herein report our experience with a 45-year-old patient with end-stage liver disease secondary to HCV-related cirrhosis and portal hypertension. While on the waiting list for liver transplant, he underwent endoscopic esophageal variceal ligation and cholecystectomy for cholelithiasis. Preoperatively, a CT scan demonstrated a 3b PVT, with partial thrombosis of the SMV and a complete thrombosis of the right branch of the portal vein (Figure 1), along with diffuse portosystemic shunts (Figure 1). During the hepatectomy for LT, a complete thrombosis of the PV and retropancreatic SMV was found, with no flow directed to the liver. No splenorenal shunts were observed in the preoperative imaging neither intraoperatively (Figure 1). To restore an adequate portal flow to the liver, an iliac vein allograft from the same donor was brought into the operative field. The iliac vein graft was anastomosed end to side to the recipient right colic vein with a 6-0 monofilament running suture, under clamp control. After the anastomosis was completed and the clamp on the colic vein released, the allograft filled promptly. Then, it was passed anteriorly to the pancreas and behind the pylorus, and after evaluating the adequate length of the graft, an end-to-end anastomosis was sewn with a 6-0 monofilament running suture between the iliac vein graft and the donor PV (Figures 2 and 3). The transplantation was completed with the rearterialization of the hepatic allograft and reconstruction of the bile duct with a Roux-en-Y technique. The cavocavostomy has already been sewn end to side before constructing the jump graft. At the end of the procedure, good portal, arterial, and vein flows were assessed at the Doppler ultrasound test (portal inflow 20 cm/sec). The patient recovered well, with a progressive improvement of the general conditions, and was finally discharged on p.o.d. 14. For the first month after the surgery, enoxaparin 4000 UI (low-molecular-weight heparin) was administered as anticoagulation therapy, and then, acetylsalicylic acid 100 mg/day was prescribed. After 12 months of follow-up, the patient appeared in good clinical conditions, with regular intrahepatic blood flows and laboratory tests in range. No anastomotic defects were found at the postoperative CT scan 10 months after the surgery (Figure 4).

### 3.2. Literature Review

To reconstitute the portal vein flow to the liver graft, Pinna and colleagues described in 1996 a novel technique of anastomosing two iliac venous allografts separately to two jejunal branches of the SMV in a patient with both portal vein and SMV thrombosis [5].

In 2009, Sato and colleagues reported 4 cases of PVT treated with a superficial femoral vein (SFV) graft interposition during living donor LT [6]. Among those 4 patients, 2 died at 2 and 35 months after transplantation from multiple organ failure associated with multiple liver abscesses, despite a well-maintained portal inflow; 1 patient developed a recurrent thrombus after a 10-month follow-up which was treated with warfarin, and although complete occlusion of the SFV graft in this patient was suspected at 15-month follow-up CT, portal inflow was maintained adequately owing to collateral vessels at 28-month follow-up.

In 2012, Mizuno et al. reported their experience with three patients who underwent living donor LT: PVT was treated with an interpositional vascular conduit passing posterior to the pancreatic parenchyma without using a jump graft [7]. In detail, an external iliac vein (or internal jugular vein) graft was harvested and passed posterior to the pancreas for anastomosis to the cut end of the SMV. The interpositional graft vein was anastomosed to the portal vein of the liver graft and flow checked by Doppler duplex ultrasound. The authors reported a good postoperative course, and only a patient whose iliac vein was used as a conduit experienced transient swelling of the lower extremity but without long-term morbidity.

Lee et al. reported in 2014 the case of a patient with grade 4 PVT successfully treated with the use of pericholedochal plexus for portal inflow restoration [8]. In detail, they performed a direct anastomosis to the donor's PV with interrupted sutures using 5-0 Prolene sutures. One week after LT, the ultrasound examination showed a normal portal flow, and the patient recovered fully without complications. Two years later, the patient was doing well, with normal liver function.

Mori and colleagues reported a series of 282 consecutive liver donor LTs, including 48 patients (17%) with PVT [4]. Thrombectomy (or thromboendovenectomy, 30 cases collectively) and replacement of the PV trunk with vein grafts were considered in their work. Replacing strategies were as follows: replaced graft in 7 patients, jump graft between the recipient's SMV and the donor PV in 7 cases, and interposed graft between the recipient's left renal vein and the donor PV in 1 patient. Finally, 3 cases did not require surgical intervention owing to minimal thrombosis. They demonstrated that no rethrombosis followed the interposition of a jump graft, while 1 stenosis occurred; conversely, 4 cases of rethrombosis and 3 of stenosis were observed after thrombectomy.

Hwang and colleagues described in 2015 the outcomes of a patient treated with a bypass from the inferior mesenteric vein to the PV using the PTFE graft for PVT after unsuccessful mechanical thrombectomy [9]. On p.o.d. 1, the ultrasound examination revealed a normal waveform without thrombus (mean flow velocity of 34.9 cm/second). The patient underwent general anesthesia again on p.o.d. 16 for perihepatic hematoma removal, but both the PV and the PTFE graft were intact. After 2 years from LT, a normal portal inflow was still demonstrable.

Recently, Pinheiro described a reconstruction with the right gastroepiploic vein (RGEV) [10]. The allograft SMV was brought anteriorly to the pancreas and behind the distal antrum and pylorus, and the RGEV was brought through a tunnel into the transverse colon, performing an end-to-end portal vein to RGEV anastomoses. Six months after LT, the patients was already doing well with good liver function.

## 4. Discussion

To our knowledge, this is the first report in literature of portal extra-anatomic jump graft from the right colic vein with donor iliac vein interposition. In an emergent condition like a complete portomesenteric thrombosis, a flexible and creative approach with an understanding of the mesenteric venous anatomy and collaterals may save the patient from a multivisceral transplantation [11]. Moreover, the technique should be relatively simple and reproducible. Other techniques like cavoportal hemitransposition (CPHT) and renoportal anastomosis (RPA), although feasible, are related to increased postoperative morbidities [12]. In particular, the most frequent complications reported in literature after CPHT and RPA are ascites, renal dysfunction, and variceal bleeding, with an overall mortality of 26%. The persistence of those typically portal hypertension-related symptoms is consistent with the fact that CPHT turns a condition of diffuse PVT with liver disease into a condition of diffuse PVT in patients with a healthy liver [12]. As reported by Rodriguez-Castro and colleagues in their interesting review, approximately 50% of patients who underwent CPHT suffer from residual portal hypertensive complications. In detail, among the 49 patients with CPHT identified in their literature research, 20% had episodes of variceal bleeding, 58% had persistent ascites, and 26% presented renal dysfunction after LT [13]. As reported in literature, RPA should be the strategy of choice when the patient has a preexistent spontaneous or surgically constructed splenorenal shunt prior to LT [12]. As a matter of fact, it is more reproducible than CPHT and preserves the physiological retrohepatic inferior vena cava [14]. In our case, the right colic vein was identified in the preoperative workup as a possible solution for the construction of a physiological inflow to the liver, due to its 10 mm diameter. Moreover, the absence of splenorenal shunts was the reason for the exclusion of a renoportal reconstruction. However, only the intraoperative evaluation of an appropriate flow eventually allowed the use of this vessel. We may hypothesize that other mesenteric vessels could have been used as described in literature. A surgically constructed splenorenal shunt with a renoportal anastomosis could be another option, as well as a CPHT. However, nonanatomical and nonphysiological reconstructions should be avoided to obtain long-term liver function. As reported by Hibi and colleagues, patients who underwent a nonphysiological reconstruction (i.e., CPHT, RPA, and arterialization) showed not only higher morbidity rates but also shorter 1-, 5-, and 10-year overall survival compared to patients with PVT treated with a physiological approach (*p*=0.043) [3].

## 5. Conclusions

Although this is the first experience of extra-anatomic jump graft from the right colic vein with donor iliac vein interposition, and further studies are needed to state the actual risk and the morbidity rate after this procedure, our technical innovation may represent a valid and safe alternative to the CPHT. It provides a proper flow restoration and reproduces a physiological setting, while avoiding the complications related to the cavoportal shunt. Notably, the demonstration of a liver-directed inflow is crucial when choosing a mesenteric vein for the anastomosis with the jump graft. We believe that the reconstitution of liver portal inflow should be obtained with the most physiological approach possible and considering long-term liver function.
